# Supplementary material for: Znhit1 controls intestinal stem cell maintenance by regulating H2A.Z incorporation
Source: Nat Commun. 2019 Mar 6;10:1071. doi: 10.1038/s41467-019-09060-w (PMC6403214; doi:10.1038/s41467-019-09060-w)
Supplement: Supplementary file 1 — Supplementary Information [file 41467_2019_9060_MOESM1_ESM.pdf]

Supplementary Information

Znhit1 controls intestinal stem cell maintenance by regulating H2A.Z incorporation

Zhao et al.

Supplementary Figure 1-12

Supplementary Table 1-3

Supplementary Figure 1

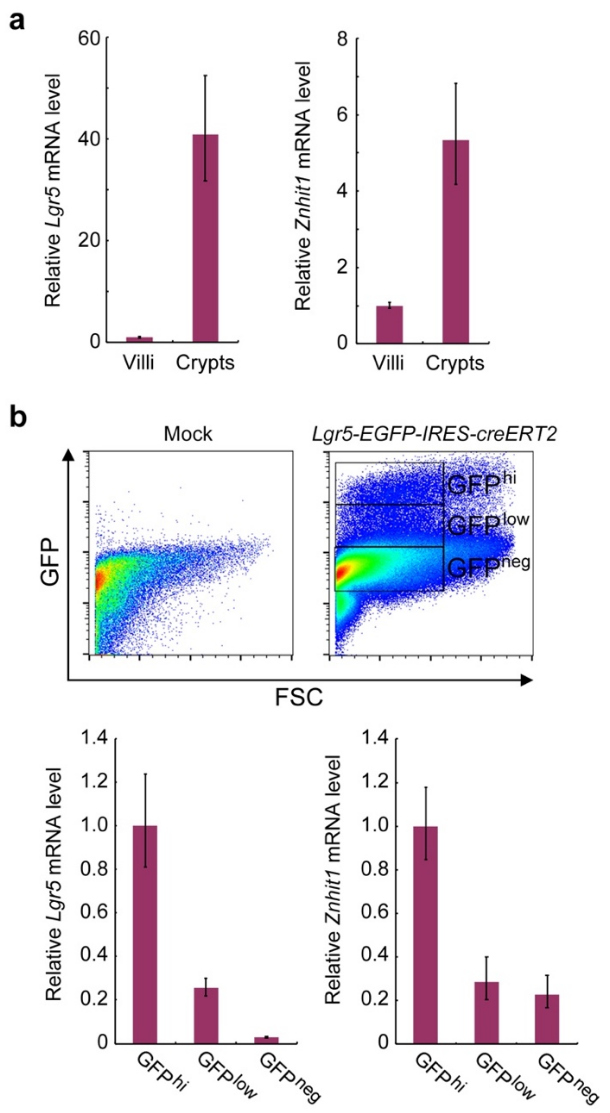

Supplementary Figure 1 | Expression pattern of Znhit1 in intestinal epithelium. (**a**) Intestinal villi and crypts were mechanically isolated from 8-week-old C57BL/6 mice then subjected to qRT-PCR to examine the expression of *Lgr5* and *Znhit1*. (**b**) The crypts harvested from 8-week-old *Lgr5-EGFP-IRES-creERT2* mice were dissociated into single cells then subjected to FACS. qRT-PCR was employed to examine the expression of *Lgr5* and *Znhit1* in Lgr5+ ISCs (GFP^{hi}), daughter progenitor cells (GFP^{low}) and other crypt cells (GFP^{neg}). Histone H3 was used as an internal control. The statistical data represent mean±s.d. (n=3 mice).

Supplementary Figure 2

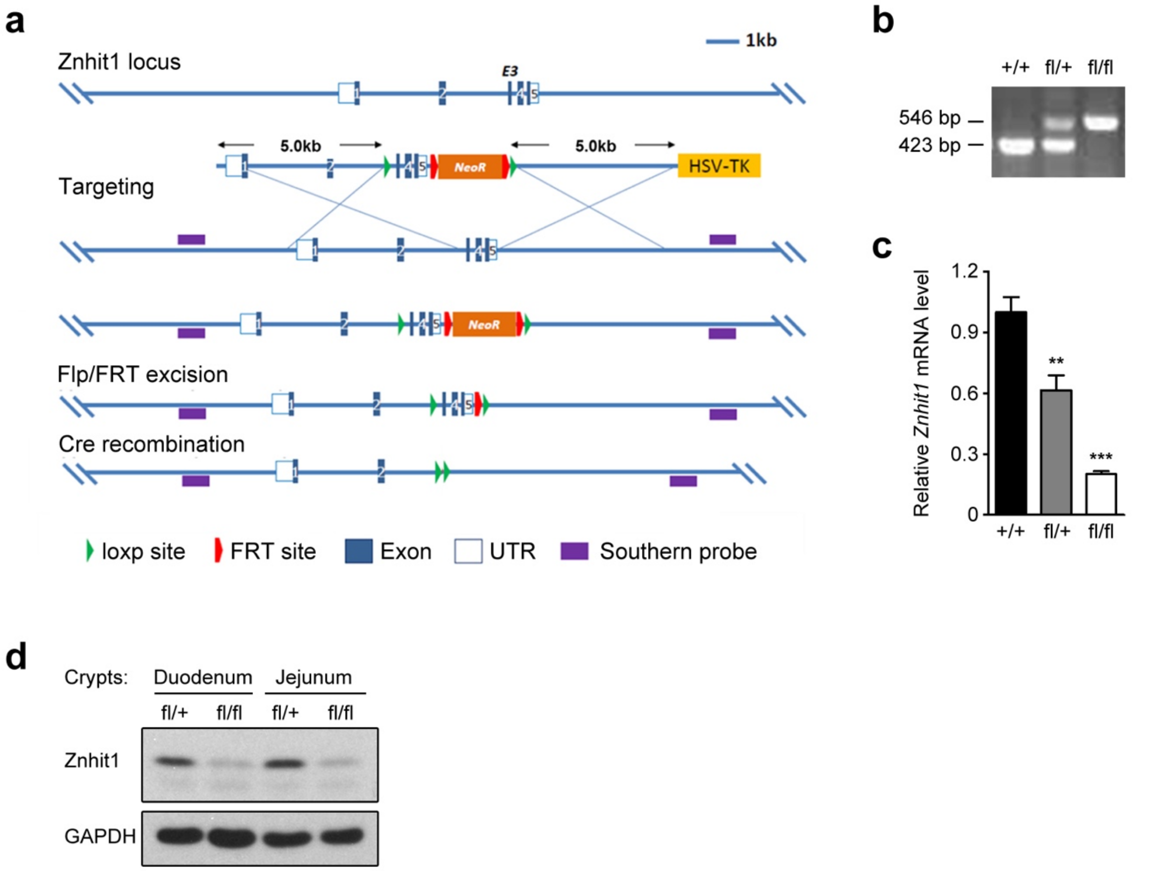

Supplementary Figure 2 | Tissue-specific Znhit1 deletion in intestinal epithelium. (**a**) Targeting strategy of *Znhit1*$^{fl/fl}$ mice (**b**) Genotyping of *Villin cre* (+/+), *Znhit1*$^{fl/+}$; *Villin-cre* (fl/+) and *Znhit1*$^{fl/fl}$; *Villin-cre* (fl/fl) mice. 423bp: wildtype allele. 546bp: floxed allele. (**c**) Intestine were harvested from *Villin cre* (+/+), *Znhit1*$^{fl/+}$; *Villin-cre* (fl/+) and *Znhit1*$^{fl/fl}$; *Villin-cre* (fl/fl) mice at P0 to examine *Znhit1* expression using qRT-PCR. Histone H3 was used as an internal control. The statistical data represent mean±s.d. (n=3 mice per genotype). Student's *t*-test: *** indicates P<0.001. ** indicates P<0.01. (**d**) Intestinal crypts were isolated from *Znhit1*$^{fl/+}$; *Villin-cre* (fl/+) and *Znhit1*$^{fl/fl}$; *Villin-cre* (fl/fl) mice at P9 for immunoblotting with the indicated antibodies. GAPDH served as a loading control.

Supplementary Figure 3

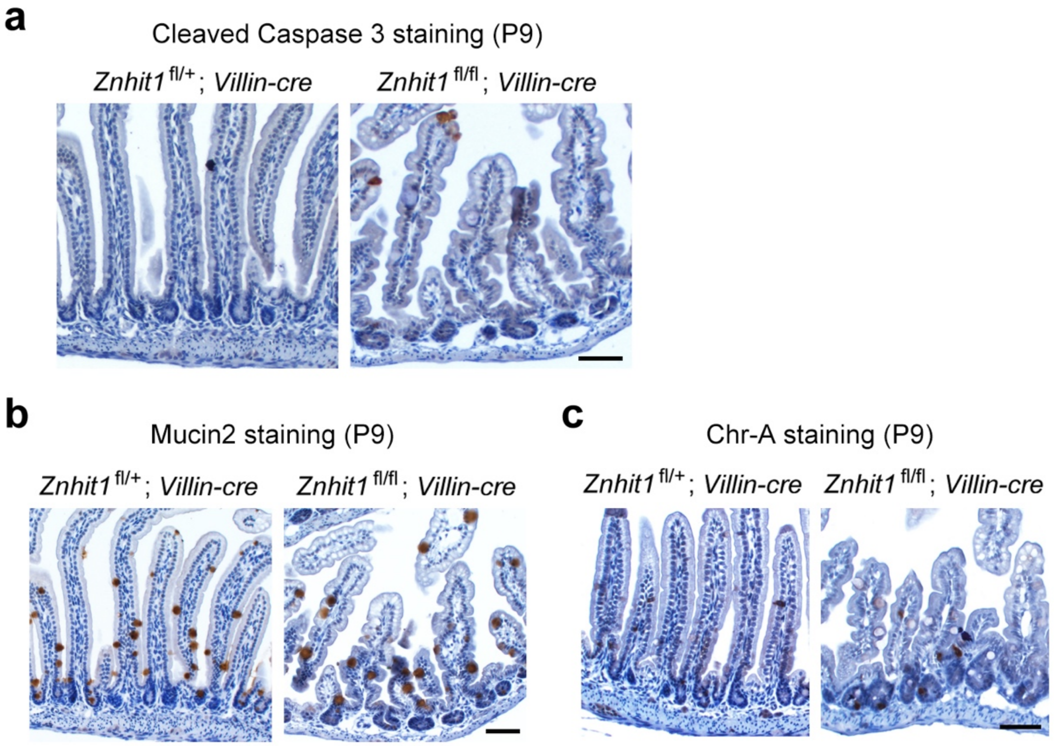

Supplementary Figure 3 | Znhit1 deletion in intestinal epithelium has no effect on crypt cell death or villus cell terminal differentiation. Cleaved Caspase 3, Mucin2 and Chr-A staining of intestinal sections from *Znhit1*$^{fl/+}$; *Villin-cre* and *Znhit1*$^{fl/fl}$; *Villin-cre* mice at P9. Scale bar, 50 µm.

Supplementary Figure 4

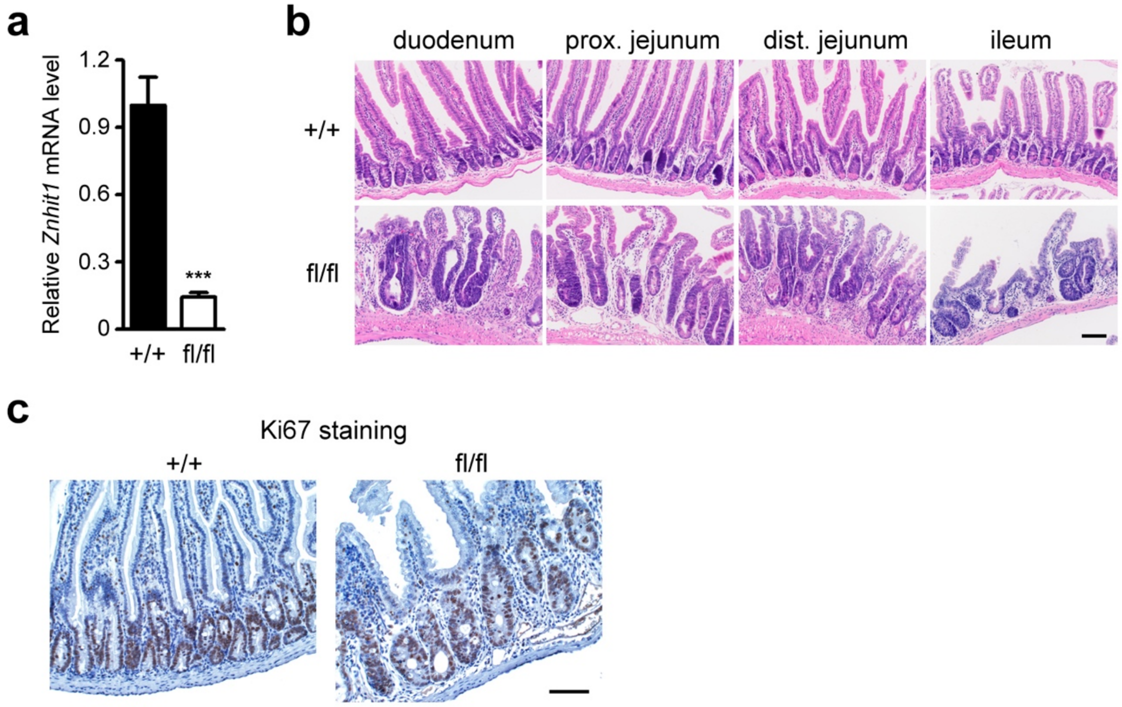

Supplementary Figure 4 | Inducible knockout of Znhit1 in intestinal epithelium. (**a**) Eight-week-old *Villin-creERT* (+/+) and *Znhit1*$^{fl/fl}$; *Villin-creERT* (fl/fl) mice were daily injected with tamoxifen for 4 days followed by 7-day waiting period. Intestinal crypts were harvested for qRT-PCR to examine *Znhit1* expression. Histone H3 was used as an internal control. The statistical data represent mean±s.d. (n=3 mice per genotype). Student's *t*-test: *** indicates P<0.001. (**b**) Paraffin embedded intestine tissues of *Villin-creERT* (+/+) and *Znhit1*$^{fl/fl}$; *Villin-creERT* (fl/fl) mice following tamoxifen treatment were stained with haematoxylin and eosin. Scale bar, 100 µm. (**c**) Ki67 staining of intestinal sections from *Villin-creERT* (+/+) and *Znhit1*$^{fl/fl}$; *Villin-creERT* (fl/fl) mice following tamoxifen treatment. Scale bar, 50 µm. A representative result of three independent experiments was shown.

Supplementary Figure 5

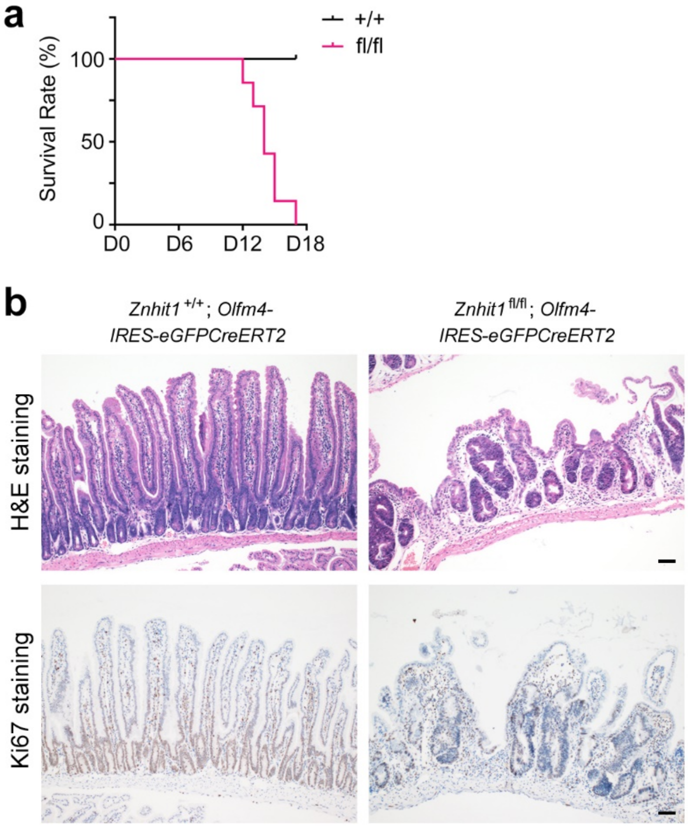

Supplementary Figure 5 | Inducible knockout of Znhit1 Lgr5+ ISCs. Eight-week-old *Znhit1*$^{+/+}$; *Olfm4-IRES-eGFPCreERT2* (+/+) and *Znhit1*$^{fl/fl}$; *Olfm4-IRES-eGFPCreERT2* (fl/fl) mice were daily injected with tamoxifen for 4 days. (**a**) Kaplan–Meier survival curves following tamoxifen administration (n=7 mice per genotype). (**b**) Embedded intestine tissues (D14-16) were subjected to H&E and Ki67 staining. A representative result of three independent experiments was shown. Scale bar, 100 µm.

Supplementary Figure 6

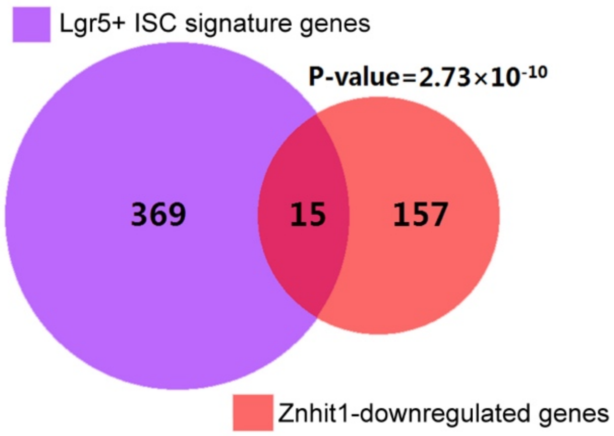

Gene ID	Log2FC
Clic6	-3.14
Olfm4	-2.91
1700040L02Rik	-2.85
Tnfsf10	-2.79
Esrrg	-2.61
Lgr5	-2.19
Dach1	-2.17
Nrtn	-2.15
Fam171a1	-2.14
Scn2b	-2.12
Ppp1r9a	-2.09
Slc27a2	-1.93
Hdac11	-1.93
Acvr2b	-1.80
Cdo1	-1.80

Supplementary Figure 6 | Znhit1 deletion induces a significant downregulation of 15 Lgr5+ ISC signature genes. Venn diagram showing the overlap between Lgr5+ ISC signature genes TSS and Znhit1-downregulated genes. The significance was evaluated by fisher's exact test.

Supplementary Figure 7

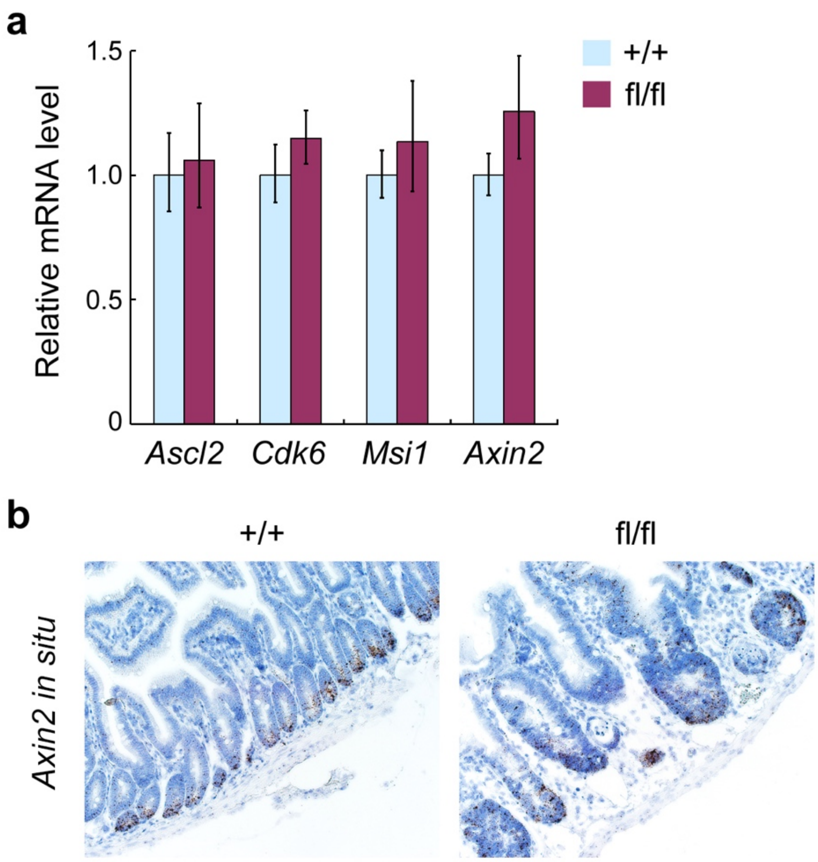

Supplementary Figure 7 | Znhit1 deficiency has no obvious effect on the expression of *Ascl2*, *Cdk6*, *Msi1* or *Axin2*. (**a**) Eight-week-old *Villin-creERT* (+/+) and *Znhit1*$^{fl/fl}$*; Villin-creERT* (fl/fl) mice were daily injected with tamoxifen for 4 days followed by 7-day waiting period. Intestinal crypts were harvested for qRT-PCR to examine the expression of indicated genes. Histone H3 was used as an internal control. The statistical data represent mean±s.d. (n=3 mice per genotype). (**b**) *Axin2 in situ* was performed in intestinal sections from *Villin-creERT* (+/+) and *Znhit1*$^{fl/fl}$; *Villin-creERT* (fl/fl) mice following tamoxifen treatment. A representative result of three independent experiments was shown.

Supplementary Figure 8

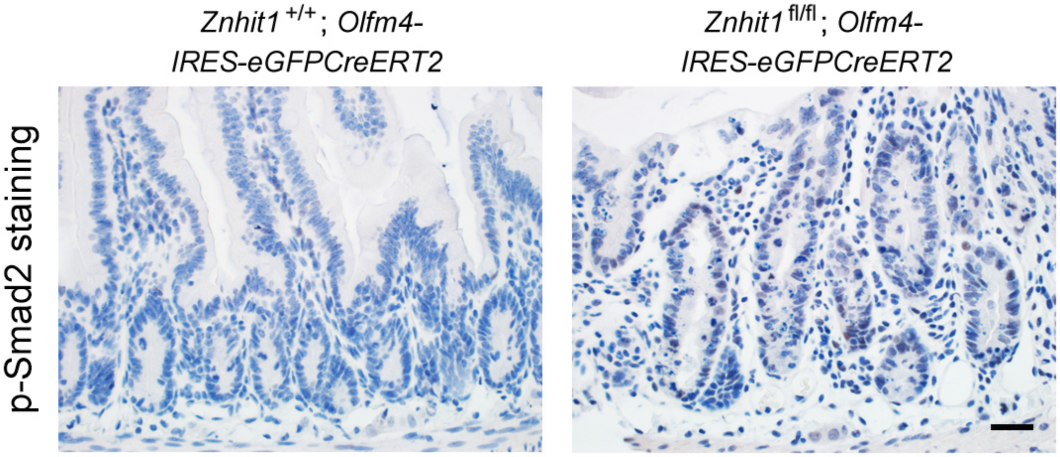

Supplementary Figure 8 | Lgr5+ ISC specific Znhit1 deletion leads to TGF-β activation in crypts. *Znhit1*$^{+/+}$; *Olfm4-IRES-eGFPCreERT2* and *Znhit1*$^{fl/fl}$; *Olfm4-IRES-eGFPCreERT2* mice were daily injected with tamoxifen for 4 days followed by 7-day waiting period. Then, embedded intestine tissues were subjected to phospho-Smad2 staining. A representative result of three independent experiments was shown. Scale bar, 50 μm.

Supplementary Figure 9

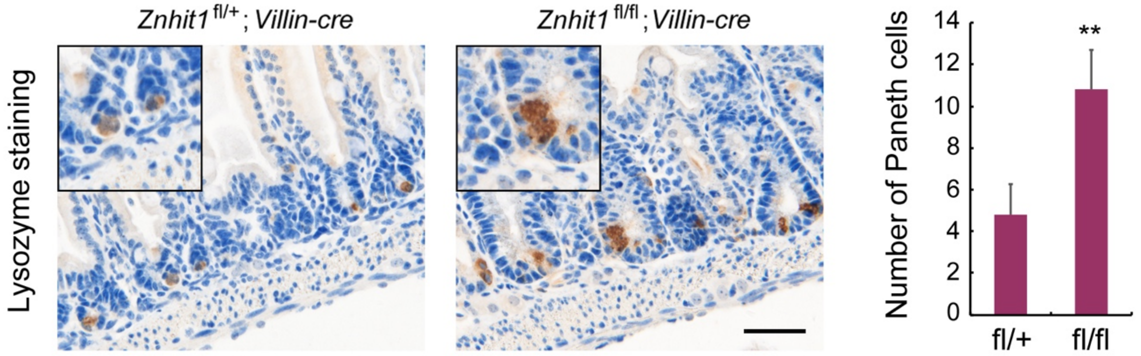

Supplementary Figure 9 | Znhit1 deficiency promotes the differentiation of Paneth cells. Lysozyme staining of intestinal sections from *Znhit1*$^{fl/+}$*; Villin-cre* and *Znhit1*$^{fl/fl}$*; Villin-cre* mice at P15. A representative result of three independent experiments was shown. Scale bar, 100 μm. The Lysozyme+ cells were quantified, and the data represent mean±s.d. (n=3 mice per genotype). Student's *t*-test: ** indicates P<0.01.

Supplementary Figure 10

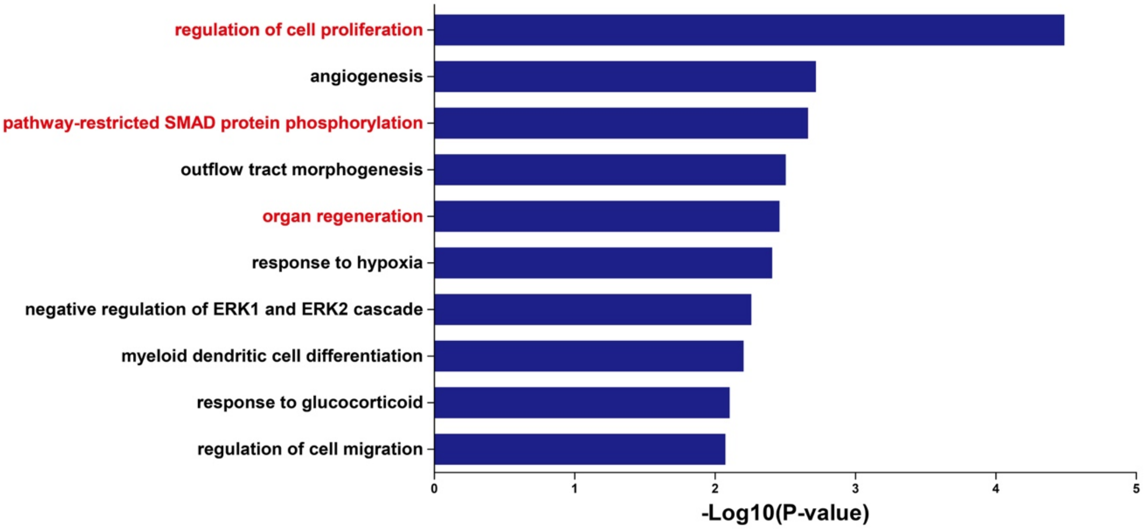

Supplementary Figure 10 | GO biological function enrichment analysis of 107 Znhit1-regulated genes with TSS H2A.Z binding.

Supplementary Figure 11

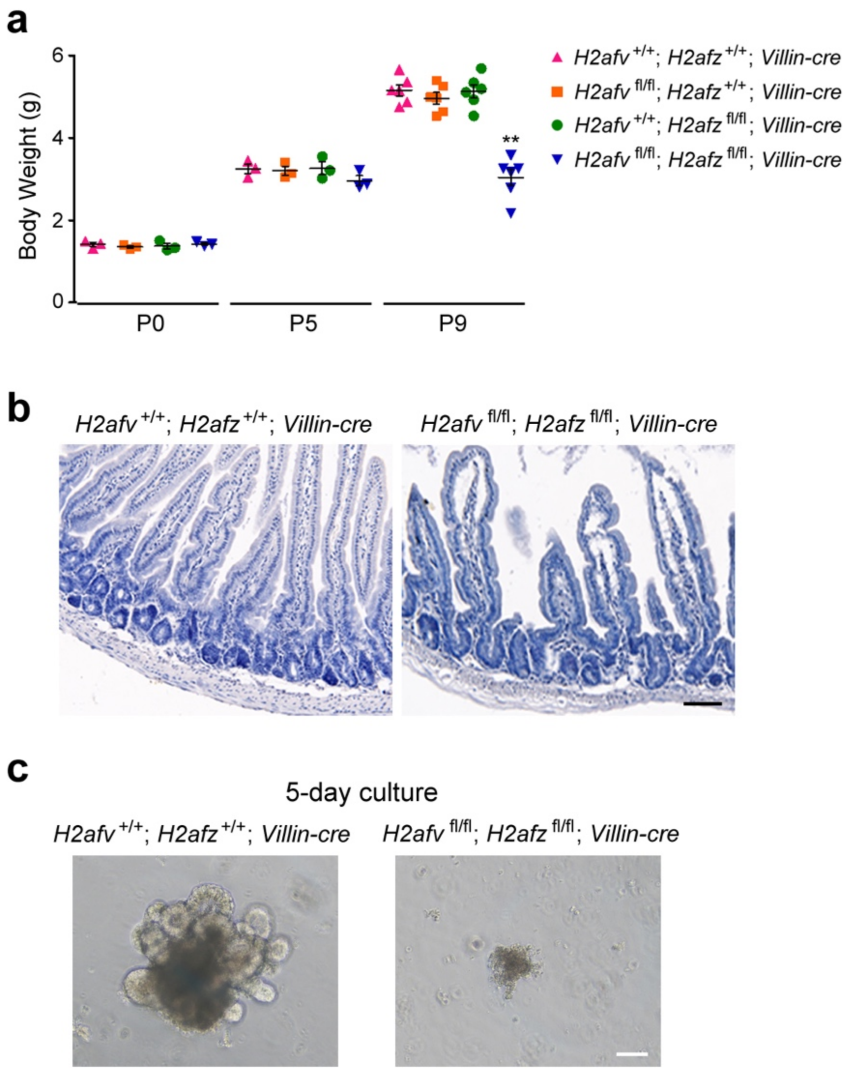

Supplementary Figure 11 | H2A.Z deletion mimics Znhit1-deficient phenotype. (**a**) Body weights of *H2afv*$^{+/+}$; *H2afz*$^{+/+}$; *Villin-cre*, *H2afv*$^{fl/fl}$; *H2afz*$^{+/+}$; *Villin-cre*, *H2afv*$^{+/+}$; *H2afz*$^{fl/fl}$; *Villin-cre* and *H2afv*$^{fl/fl}$; *H2afz*$^{fl/fl}$; *Villin-cre* mice at indicated time. The data represent mean±s.d. (n=5 mice per group). Kruskal-Wallis' H test: ** indicates P<0.01. (**b**) Intestine sections of *H2afv*$^{+/+}$; *H2afz*$^{+/+}$; *Villin-cre* and *H2afv*$^{fl/fl}$; *H2afz*$^{fl/fl}$; *Villin-cre* mice. A representative result of three independent experiments was shown. Scale bar, 100 μm. (**c**) Intestinal crypts were isolated from *H2afv*$^{+/+}$; *H2afz*$^{+/+}$; *Villin-cre* and *H2afv*$^{fl/fl}$; *H2afz*$^{fl/fl}$; *Villin-cre* mice at P9, embedded in Matrigel (100 crypts per well) and cultured for 5 days. Scale bar, 50 μm.

Supplementary Figure 12

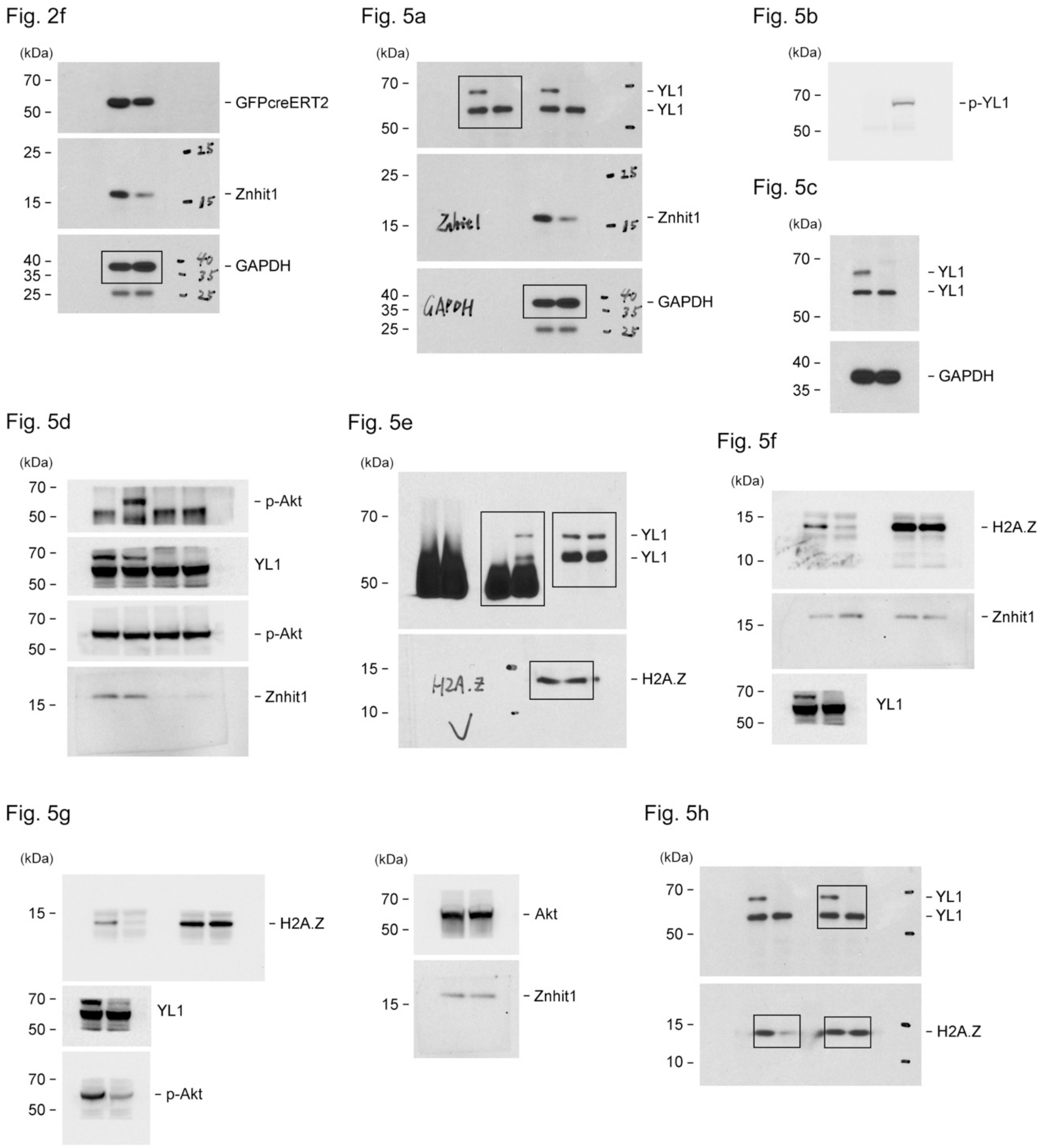

Supplementary Figure 12 | Full immunoblots.

Supplementary Table 1｜107 genes Znhit1-regulated genes with TSS H2A.Z binding.

Gene ID	log2FoldChange	P-value
Ctsl	3.45	8.68E-144
Areg	3.15	6.18E-137
Pdlim7	3.62	4.91E-125
Cyp4v3	-2.69	4.43E-118
Hmox1	4.28	3.25E-103
Cbr3	4.76	8.99E-103
Tgfbr2	2.26	2.15E-85
Psat1	2.26	7.71E-85
Ppl	2.39	1.09E-77
Emp2	2.75	3.34E-77
Abcb1b	3.32	2.81E-73
Crip2	3.58	2.40E-62
Adm2	5.43	1.64E-61
Plbd1	-2.10	3.09E-60
Dusp3	2.57	6.60E-58
Avpi1	2.34	2.78E-56
Rab11fip5	3.82	2.01E-55
Spry4	3.19	4.85E-54
Htra1	3.65	5.29E-54
Smim1	3.81	2.12E-53
Dpysl3	3.15	8.44E-52
Ptrf	3.38	3.06E-51
Trib3	2.73	3.72E-51
Plau	4.32	3.56E-50
Gcnt1	3.01	1.19E-49
Pcca	-2.11	4.22E-49
Arg2	-2.18	4.93E-49
Lamc3	3.40	2.92E-48
Svip	-2.31	4.92E-48
Ereg	2.57	1.60E-47
Tubb2a	1.81	2.19E-44
Car4	-1.80	2.30E-43
Pmm1	1.89	2.42E-43
Gabarapl1	1.88	6.55E-43
Hk1	2.34	8.53E-43
Scrn2	-1.77	1.59E-40

Fam129a	2.38	4.06E-39
Fbp1	-1.95	1.42E-38
Ephx1	2.50	2.58E-38
Pik3r3	1.86	1.47E-37
Tnfrsf12a	3.03	9.52E-37
Itgb3	3.20	2.27E-36
Trf	3.17	4.66E-36
Icam1	2.59	2.25E-32
Fam20a	-1.70	2.32E-32
Rab19	-1.98	2.39E-31
Plxnd1	2.01	9.85E-31
Dusp1	1.98	4.76E-30
Opn3	2.19	2.99E-29
Setd4	1.87	4.92E-29
Dennd5a	1.84	2.02E-28
Cadm4	2.44	2.29E-28
Ltbp2	4.16	6.30E-28
Gpr157	-1.81	7.95E-28
Rgcc	1.98	8.93E-28
Fzd1	2.85	1.99E-27
AI427809	-2.70	1.97E-26
Chac1	2.43	2.14E-26
Plk2	4.42	3.96E-26
Nkain4	3.43	8.42E-25
Ffar4	1.97	2.49E-24
Smtnl2	3.01	2.86E-24
Mdfi	5.31	2.97E-24
Gpsm1	1.96	5.56E-24
Vwce	-2.33	1.10E-23
Tgfbr3	2.97	1.16E-23
Emc9	-1.88	3.78E-22
Slc35e4	2.81	1.58E-20
Timp3	3.24	2.61E-20
Macrod1	-1.81	4.81E-20
Npr1	2.91	6.33E-20
Klf2	2.45	6.81E-20
Slc27a2	-1.93	6.43E-19
Cdkn1c	1.86	3.65E-18
S100a14	1.97	7.49E-18
Arhgef37	2.73	1.63E-17
8430419L09Rik	-2.49	6.02E-15

Fa2h	4.23	8.63E-15
Tcea3	-2.50	3.34E-14
Etv4	2.33	3.56E-14
Cxcl2	5.48	3.60E-14
Tnfsf9	2.60	1.54E-13
Pyroxd2	-1.96	3.78E-12
Trp53inp1	2.09	3.86E-12
Btg3	1.88	8.63E-12
Plcd3	3.28	2.19E-11
Herc6	-1.86	9.06E-11
Ajuba	2.96	1.32E-10
Tnfaip8l3	-1.81	1.34E-10
Mal	3.94	1.37E-10
Stk32c	2.24	1.47E-10
Cercam	2.77	1.20E-09
Tgfb1	1.75	1.23E-09
Cxcl1	4.13	1.70E-09
Esrrg	-2.61	2.57E-09
Gsap	2.97	7.83E-09
Ppp1r9a	-2.09	2.78E-08
Rasl11a	1.74	3.00E-08
Cxcl5	4.39	6.78E-08
Lyplal1	-1.79	1.60E-06
Sh3bp5	1.84	2.03E-05
Gng11	-1.72	2.22E-05
Ifit2	-1.93	3.57E-05
1700003E16Rik	-2.73	3.57E-04
Car11	3.95	5.76E-04
Lgr5	-2.19	1.51E-03
Clic6	-3.14	1.54E-03

Supplementary Table 2 | Primers for RT-qPCR.

Quantitiative RT-PCR primers	Forward (5'-3')	Reverse (5'-3')
H3	TGTGGCCCTCCGTGAAATC	GGCATAATTGTTACACGTTTGGC
Znhit1	TGGGCAAGAGGCTACCTCA	CAGATGCACTCAGGTTCTGCT
Lgr5	CGGGACCTTGAAGATTTCCT	GATTCGGATCAGCCAGCTAC
Ascl2	TGCCGCACCAGAACTCGTAG	ACTCCAGACGAGGTGGGCAT
Olfm4	CAGCCACTTTCCAATTTCACTG	GCTGGACATACTCCTTCACCTTA
Clic6	CTCTGGGTTAGACTCTCAGGG	GGTGCCTCTGTGTCCATGTT
Dach1	CCTGGGAAACCCGTGTACTC	AGATCCACCATTTTGCACTCATT
Esrrg	AAGATCGACACATTGATTCCAGC	CATGGTTGAACTGTAACTCCCAC
Scn2b	CGGAGCATGGAAGTCACAG	CTGCTTGTGGTTCACGGTGTA
Tgfb1	CTCCCGTGGCTTCTAGTGC	GCCTTAGTTTGGACAGGATCTG
Tgfbr2	CCGCTGCATATCGTCCTGTG	AGTGGATGGATGGTCCTATTACA
Pla2g2e	CCAGTGGACGAGACGGATTG	AGCAGCTCTCTTGTCACACTC
Lyz2	ATGGAATGGCTGGCTACTATGG	ACCAGTATCGGCTATTGATCTGA
Hopx	AGGAGCAGACGCAGAAATG	GAAACATCAAAACAGCCTGGG
H2afv	GCTAAGGCGGTGTCTCGTTC	TGTGGTGCGAGTCTTCAAGTG
H2afz	CCAAGACAAAGGCGGTTTCC	TCCTGCCAACTCAAGTACCTC
Cdk6	GGCGTACCCACAGAAACCATA	AGGTAAGGGCCATCTGAAAACT
Msi1	TAAAGTGCTGGCGCAATCG	TCTTCGTCCGAGTGACCATCT
Axin2	GCTCCAGAAGATCACAAAGAGC	AGCTTTGAGCCTTCAGCATC

Supplementary Table 3 | Primers for ChIP-qPCR.

ChIP-qPCR primers	Forward (5'-3')	Reverse (5'-3')
Negative region	CCCTCTACAGAACCACC	TCCTTCATTCCCACATC
Lgr5	GGTGAAGACGCTGAGGTTGG	CCTCTACAGGCTCCCTGCTCT
Clic6	CTCACCCTGAGCAGCGTCG	CTCCTGGTCCCTCGATTGTC
Tgfbr2	CGAGATGGCAAAGCTGAGGA	CCGGAAAGGGAAGTTTAAGAAGT
Tgfb1	GCACTGCGCTGTCTCGCAAGGA	TTTGTGGCTCCCGAGGGCTGGT
