# Supplementary material for: Znhit1 controls intestinal stem cell maintenance by regulating H2A.Z incorporation
Source: Nat Commun. 2019 Mar 6;10:1071. doi: 10.1038/s41467-019-09060-w (PMC6403214; doi:10.1038/s41467-019-09060-w)
Supplement: Supplementary file 3 — Reporting Summary [file 41467_2019_9060_MOESM3_ESM.pdf]

nature research

Corresponding author(s): Bing Zhao and Xinhua Lin

Last updated by author(s): Jan 28, 2019

# Reporting Summary

Nature Research wishes to improve the reproducibility of the work that we publish. This form provides structure for consistency and transparency in reporting. For further information on Nature Research policies, see Authors & Referees and the Editorial Policy Checklist.

## Statistics

For all statistical analyses, confirm that the following items are present in the figure legend, table legend, main text, or Methods section.

| n/a | Confirmed | |
|---|---|---|
| ☐ | ☒ | The exact sample size (*n*) for each experimental group/condition, given as a discrete number and unit of measurement |
| ☐ | ☒ | A statement on whether measurements were taken from distinct samples or whether the same sample was measured repeatedly |
| ☐ | ☒ | The statistical test(s) used AND whether they are one- or two-sided<br>*Only common tests should be described solely by name; describe more complex techniques in the Methods section.* |
| ☒ | ☐ | A description of all covariates tested |
| ☐ | ☒ | A description of any assumptions or corrections, such as tests of normality and adjustment for multiple comparisons |
| ☐ | ☒ | A full description of the statistical parameters including central tendency (e.g. means) or other basic estimates (e.g. regression coefficient) AND variation (e.g. standard deviation) or associated estimates of uncertainty (e.g. confidence intervals) |
| ☐ | ☒ | For null hypothesis testing, the test statistic (e.g. *F*, *t*, *r*) with confidence intervals, effect sizes, degrees of freedom and *P* value noted<br>*Give P values as exact values whenever suitable.* |
| ☒ | ☐ | For Bayesian analysis, information on the choice of priors and Markov chain Monte Carlo settings |
| ☐ | ☒ | For hierarchical and complex designs, identification of the appropriate level for tests and full reporting of outcomes |
| ☐ | ☒ | Estimates of effect sizes (e.g. Cohen's *d*, Pearson's *r*), indicating how they were calculated |

*Our web collection on statistics for biologists contains articles on many of the points above.*

## Software and code

Policy information about availability of computer code

| | |
|---|---|
| Data collection | Beckman Coulter MoFlo XDP was operated using Summit Software version 5.2. FlowJo (Treestar) was used to format FACS plots.<br>For RNA-seq, RNA from freshly isolated intestinal crypts was converted into cDNA libraries using the Ovation® RNA-Seq System V2 kit (NuGEN). High-throughput sequencing was performed using Illumina HiSeq X10 for 3 biological replicates.<br>For Chip-seq, the immunoprecipitated chromatin was subjected to library construction and sequencing on Illumina HiSeq 2500 by BerryGenomics. |
| Data analysis | The RNA-seq data was mapped to mm10 genome by TopHat v1.4.1 with no more than 2 mismatches, and then only the uniquely mapped reads were used to estimate the expression values in gene level by RPKM. Statistical significant test of differentially expressed genes was performed by DEseq with R. Hierarchical clustering of log10-transformed RPKMs was generated by Cluster 3.0 and visualized by Java TreeView.<br>For ChIP-seq, sequencing reads were mapped against the reference genome (mm10) using Bowtie v1.1.1 and peaks detection was done by MACS with default cutoff. Peaks were assigned to the nearest genes using Homer. |

For manuscripts utilizing custom algorithms or software that are central to the research but not yet described in published literature, software must be made available to editors/reviewers. We strongly encourage code deposition in a community repository (e.g. GitHub). See the Nature Research guidelines for submitting code & software for further information.

## Data

Policy information about availability of data

All manuscripts must include a data availability statement. This statement should provide the following information, where applicable:

- Accession codes, unique identifiers, or web links for publicly available datasets
- A list of figures that have associated raw data
- A description of any restrictions on data availability

The raw NGS data were deposited to the NCBI SRA database under accession number SRP148616 (RNA-seq data) and SRP148519 (ChIP-seq data). The data will be

released upon publication. All other data of this study are available from the corresponding authors upon reasonable request.

# Field-specific reporting

Please select the one below that is the best fit for your research. If you are not sure, read the appropriate sections before making your selection.

☒ Life sciences ☐ Behavioural & social sciences ☐ Ecological, evolutionary & environmental sciences

For a reference copy of the document with all sections, see nature.com/documents/nr-reporting-summary-flat.pdf

# Life sciences study design

All studies must disclose on these points even when the disclosure is negative.

| | |
|---|---|
| Sample size | For each experiment listed in the manuscript, the sample sizes are as follows:<br>1. For mice body weight comparison, each group contains 3 or 5 mice at the indicated time point.<br>2. For mice survival rate comparison, each group contains 7, 9 or 19 mice.<br>3. For immunohistology experiments, at least 3 pairs of mice tissue were processed. For each staining, at least 15 images were taken randomly, 1 representative image were shown in the figure.<br>4. For organoid culture, intestinal crypts were isolated from at least 5 pairs of mice.<br>5. For rt-PCR experiments, RNA were isolated from 3 pairs of mice, each RNA sample was loaded triplicates on the reading plate.<br>6. For RNA-seq, high-throughput sequencing was performed using Illumina HiSeq X10 for 3 biological replicates.<br>7. For western blotting analysis, the experiments were repeated 3 times from protein preparation and one representative blot was shown in the figures. |
| Data exclusions | No data were excluded from the analyses. |
| Replication | All attempts at replication were successful. |
| Randomization | All knockout mice were compared with their littermates. |
| Blinding | Images were taken by person who was blinded to group allocation. |

# Reporting for specific materials, systems and methods

We require information from authors about some types of materials, experimental systems and methods used in many studies. Here, indicate whether each material, system or method listed is relevant to your study. If you are not sure if a list item applies to your research, read the appropriate section before selecting a response.

## Materials & experimental systems

| n/a | Involved in the study | |
|---|---|---|
| ☐ | ☒ | Antibodies |
| ☒ | ☐ | Eukaryotic cell lines |
| ☒ | ☐ | Palaeontology |
| ☐ | ☒ | Animals and other organisms |
| ☒ | ☐ | Human research participants |
| ☒ | ☐ | Clinical data |

## Methods

| n/a | Involved in the study | |
|---|---|---|
| ☐ | ☒ | ChIP-seq |
| ☐ | ☒ | Flow cytometry |
| ☒ | ☐ | MRI-based neuroimaging |

## Antibodies

| | |
|---|---|
| Antibodies used | Part1: IHC<br>1. anti-Ki67, BD Pharmingen, Catalog #: 550609, Clone #: B56<br>2. anti-Keratin20, Cell Signaling Technology, Catalog #: 13063, Clone #: D9Z1Z<br>3. anti-Tgfb1, Santa Cruz Biotechnology, Catalog #: sc-130348, Clone #: 3C11<br>4. anti-Tgfbr2, Santa Cruz Biotechnology, Catalog #: sc-17792, Clone #: E-6<br>5. anti-phospho-smad2(Ser245/250/255), Cell Signaling Technology, Catalog #: 18338, Clone #: E8F3R<br>6. anti-Lysozyme, Santa Cruz, Catalog #: sc-27958, Clone #:C-19<br><br>Part 2: ChIP<br>7. anti-H2A.Z, Abcam, Catalog #: ab4174<br>8. anti-H3K4me3, Cell Signaling Technology, Catalog #: 9751, Clone #: C42D8<br>9. anti-H3K27me3, Cell Signaling Technology, Catalog #: 9733<br><br>Part 3: IB, IP<br>10. anti-GFP, Clontech, Catalog #: 8372-2 |

11. anti-Znhit1, Sigma, Catalog #: HPA019043
12. anti-GAPDH, Origene, Catalog #: TA802519, Clone #: OTI2D9
13. anti-YL1, Abcam, Catalog #: ab112055
14. anti-phospho-Ser/Thr, Cell Signaling Technology, Catalog #: 9614, Clone #: 110B7E
15. anti-p-Akt, Cell Signaling Technology, Catalog #: 4060, Clone #: D9E
16. anti-H2A.Z, Abcam, Catalog #: ab4174
17. anti-Akt, Cell Signaling Technology, Catalog #: 4685, Clone #: 11E7

Validation

1. anti-Ki67, BD Pharmingen, Catalog #: 550609, Species Cross-Reactivity: H, M, R, Applications: WB, IF, IHC
2. anti-Keratin20, Cell Signaling Technology, Catalog #: 13063, Species Cross-Reactivity: H, Applications: W, IHC-P, IF-IC, F
3. anti-Tgfb1, Santa Cruz Biotechnology, Catalog #: sc-130348, Species Cross-Reactivity: mouse, rat, human, Applications: WB, IF, IHC(P)
4. anti-Tgfbr2, Santa Cruz Biotechnology, Catalog #: sc-17792, Species Cross-Reactivity: mouse, rat, human, Applications: WB, IP, IF, IHC(P), ELISA
5. anti-phospho-smad2(Ser245/250/255), Cell Signaling Technology, Catalog #: 18338, Species Cross-Reactivity: H, M, R, Applications: W, IP, ChIP, IF-IC, F
6. anti-Lysozyme, Santa Cruz, Catalog #: sc-27958, Species Cross-Reactivity: mouse, rat, human, Applications: WB, IP, IF, ELISA
7. anti-H2A.Z, Abcam, Catalog #: ab4174, Species Cross-Reactivity: Mouse, Rat, Rabbit, Chicken, Cow, Human, Arabidopsis thaliana, Zebrafish, Applications: ICC/IF, ChIP, IP, CHIPseq, WB
8. anti-H3K4me3, Cell Signaling Technology, Catalog #: 9751, Species Cross-Reactivity: H, M, R, Mk, Sc, Dm, (X, Z), Applications: W, IHC-P, IF-IC, F, ChIP, ChIP-seq
9. anti-H3K27me3, Cell Signaling Technology, Catalog #: 9733, Species Cross-Reactivity: H, M, R, Mk, (X, Z), Applications: W, IHC-P, IF-IC, ChIP, ChIP-seq, F
10. anti-GFP, Clontech, Catalog #: 8372-2, Applications: WB, IP, IF, IHC
11. anti-Znhit1, Sigma, Catalog #: HPA019043, Species Cross-Reactivity: human, mouse, rat, Applications: immunofluorescence, immunohistochemistry, western blot
12. anti-GAPDH, Origene, Catalog #: TA802519, Species Cross-Reactivity: Human, Mouse, Rat, Dog, Monkey, Applications: WB
13. anti-YL1, Abcam, Catalog #: ab112055, Species Cross-Reactivity: Human, Applications: WB, IP
14. anti-phospho-Ser/Thr, Cell Signaling Technology, Catalog #: 9614, Species Cross-Reactivity: All, Applications: W, IP, IHC-P, E-P
15. anti-p-Akt, Cell Signaling Technology, Catalog #: 4060, Species Cross-Reactivity: H, M, R, Mk, Hm, B, Dm, Z, (Pg, C, X, Dg), Applications: W, IP, IHC-P, IF-IC, F
16. anti-Akt, Cell Signaling Technology, Catalog #: 4685, Species Cross-Reactivity: H, M, R, Mk, Applications: W, IP, IHC-P, IF-IC, F

# Animals and other organisms

Policy information about studies involving animals; ARRIVE guidelines recommended for reporting animal research

Laboratory animals

Mouse, C57BL/6, male and female, from postnatal to 8-week-old, detailed as follows:
Znhit1fl/fl mice were generated by Model Animal Research Center of Nanjing University (MARC, Nanjing, China).
Villin-cre, Villin-creERT and Lgr5-EGFP-IRES-creERT2 mice were obtained from the Jackson Laboratory. Olfm4-IRES-eGFPcreERT2 mice were provided by Hans Clevers. H2afz(fl/fl)/H2afv(fl/fl) mice were obtained from RIKEN BioResource Center (RBRC05765).
All strains were backcrossed with the C57BL/6 background mice. For Cre induction, mice were intraperitoneally injected with 100 µl tamoxifen in sunflower oil at 20 mg ml-1 for 3-4 consecutive days.

Wild animals

The study did not involve wild animals.

Field-collected samples

The study did not involve samples collected from the field.

Ethics oversight

All breeding and experimental procedures were performed in accordance with the relevant guidelines and regulations and with the approval of the Institutional Animal Care and Use Committee at Institute of Zoology or Cincinnati Children's Hospital.

Note that full information on the approval of the study protocol must also be provided in the manuscript.

# ChIP-seq

## Data deposition

☒ Confirm that both raw and final processed data have been deposited in a public database such as GEO.

☒ Confirm that you have deposited or provided access to graph files (e.g. BED files) for the called peaks.

Data access links
*May remain private before publication.*

The raw NGS data were deposited to the NCBI SRA database under accession number SRP148616 (RNA-seq data) and SRP148519 (ChIP-seq data). The data will be released upon publication. All other data of this study are available from the corresponding authors upon reasonable request.

Files in database submission

For ChIP-seq, 4 NGS data files have been submitted to NCBI SRA database under accession number SRP148519:
1. flfl.clean.fastq.gz
2. flfl-Input.clean.fastq.gz
3. ++.clean.fastq.gz
4. ++-Input.clean.fastq.gz

Genome browser session
(e.g. UCSC)

no longer applicable

## Methodology

| | |
|---|---|
| Replicates | 1 biological replicate for both KO and WT mice |
| Sequencing depth | For each sample, the ChIP-seq was sequenced by Illumina Hiseq2500 with single-end with 1×50bps length. Around 33~42M (millions) reads were sequenced for each sample, respectively. Sequenced reads were aligned to reference genome (mm10) using Bowtie (v1.1.1). About 97.9%~98.7% of sequenced reads could be mapped to reference genome (mm10) with no more than 2 mismatches, meanwhile 76.8%~79.5% of sequenced reads could be uniquely mapped to reference genome (mm10). There were about 10~30 reads could be uniquely mapped to different genomic regions per 1 kb length. And we found the reads were relatively enriched in the 5'UTR-exon and TSS_up_1kb regions with Fold Change (FC) =2.23 and 1.55. Peaks calling analysis was performed by MACS with default cutoff. |
| Antibodies | Anti-H2A.Z, Abcam, Catalog #: ab4174, Species Cross-Reactivity: Mouse, Rat, Rabbit, Chicken, Cow, Human, Arabidopsis thalina, Zebrafish, Applications: ICC/IF, ChIP, IP, ChIP-seq, WB |
| Peak calling parameters | For ChIP-seq, sequencing reads were mapped against the reference genome (mm10) using Bowtie v1.1.1. For peaks calling, Both treatment and input-control data were used by MACS algorithm with pvalue < 1e-5 and above 5-fold enrichment. |
| Data quality | FastQC software was used to check the quality of raw sequencing data. After alignment, about 97.9%~98.7% of sequenced reads could be mapped to reference genome (mm10) with no more than 2 mismatches, meanwhile 76.8%~79.5% of sequenced reads could be uniquely mapped to reference genome (mm10). ChIP-seq data revealed 6,506 peaks in WT, and only 621 peaks were detected in KO by MACS algorithm with pvalue < 1e-5 and above 5-fold enrichment. |
| Software | Bowtie v1.1.1 was used to align the sequencing data to reference genome (mm10).<br>MACS algorithm was used to detect the peaks.<br>Peaks were assigned to the nearest genes using Homer. |

# Flow Cytometry

## Plots

Confirm that:

☒ The axis labels state the marker and fluorochrome used (e.g. CD4-FITC).

☒ The axis scales are clearly visible. Include numbers along axes only for bottom left plot of group (a 'group' is an analysis of identical markers).

☒ All plots are contour plots with outliers or pseudocolor plots.

☒ A numerical value for number of cells or percentage (with statistics) is provided.

## Methodology

| | |
|---|---|
| Sample preparation | For ISC enrichment, intestines of WT or Lgr5-GFP mice were excised, dissected and cleaned with cold PBS. Then intestines were cut longitudinally and washed twice with cold PBS. Villi were carefully scraped off with operating scalpel. The remaining part was cut into small pieces (5 mm) and incubated in 10 mM EDTA in PBS for 40 min on ice. After removal of EDTA, the small pieces were vigorously suspended using a 10-ml pipette with cold PBS. The supernatant, which enriched in crypts, was passed through 70 μm cell strainer (BD) and centrifuged at 600 rpm for 3 min. The pure crypts obtained were used for single Lgr5-GFPhi cells purification. |
| Instrument | Beckman Coulter MoFlo XDP |
| Software | Beckman Coulter MoFlo XDP was operated using Summit Software version 5.2. FlowJo (Treestar) was used to format FACS plots. |
| Cell population abundance | For Lgr5-GFP mice, Lgr5-GFPhi cells were grouped separately from Lgr5-GFPlow and Lgr5-GFPneg cells, and can be analyzed/ sorted directly. About 100000 cells were obtained from each mouse. |
| Gating strategy | For ISC analysis, WT mice can serve as negative controls and GFP+ cells from Lgr5-GFP mice were analyzed or enriched directly. |

☒ Tick this box to confirm that a figure exemplifying the gating strategy is provided in the Supplementary Information.
